# Dr. Charles Benjamin Knowlton

**Published:** 1917-06

**Authors:** 


**Dr. Charles Benjamin Knowlton,** Buffalo 1872, died at his home in Buffalo, May 11, aged 81. He was also a graduate in law and had not actively engaged in the practice of medicine. To many Buffalonians, he will be best remembered for his long and valuable service in the public schools, as a teacher of writing.

